# The Orthology Ontology: development and applications

**DOI:** 10.1186/s13326-016-0077-x

**Published:** 2016-06-04

**Authors:** Jesualdo Tomás Fernández-Breis, Hirokazu Chiba, María del Carmen Legaz-García, Ikuo Uchiyama

**Affiliations:** Departamento de Informática y Sistemas, Universidad de Murcia, IMIB-Arrixaca, Murcia, 30071 Spain; National Institute for Basic Biology, National Institutes of Natural Sciences, Okazaki, 444-8585 Aichi Japan

**Keywords:** Semantic web, Knowledge representation, Ontology, Comparative genomics, Orthology

## Abstract

**Background:**

Computational comparative analysis of multiple genomes provides valuable opportunities to biomedical research. In particular, orthology analysis can play a central role in comparative genomics; it guides establishing evolutionary relations among genes of organisms and allows functional inference of gene products. However, the wide variations in current orthology databases necessitate the research toward the shareability of the content that is generated by different tools and stored in different structures. Exchanging the content with other research communities requires making the meaning of the content explicit.

**Description:**

The need for a common ontology has led to the creation of the Orthology Ontology (ORTH) following the best practices in ontology construction. Here, we describe our model and major entities of the ontology that is implemented in the Web Ontology Language (OWL), followed by the assessment of the quality of the ontology and the application of the ORTH to existing orthology datasets. This shareable ontology enables the possibility to develop Linked Orthology Datasets and a meta-predictor of orthology through standardization for the representation of orthology databases. The ORTH is freely available in OWL format to all users at http://purl.org/net/orth.

**Conclusions:**

The Orthology Ontology can serve as a framework for the semantic standardization of orthology content and it will contribute to a better exploitation of orthology resources in biomedical research. The results demonstrate the feasibility of developing shareable datasets using this ontology. Further applications will maximize the usefulness of this ontology.

## Background

Owing to rapid progress in sequencing technologies, the number of genome sequences determined has significantly increased; recently, the targets of genome projects are not limited to the model organisms but include uninvestigated organisms of particular interest. In this new genomic era, the role of computational analysis is becoming increasingly important. There is an urgent need for consolidating a comprehensive foundation of comparative analysis toward effective knowledge discovery. In particular, the orthology information is a key resource; it guides establishing evolutionary histories among genes of multiple organisms and provides a basis for functional inference of gene products.

**Fig. 1:**
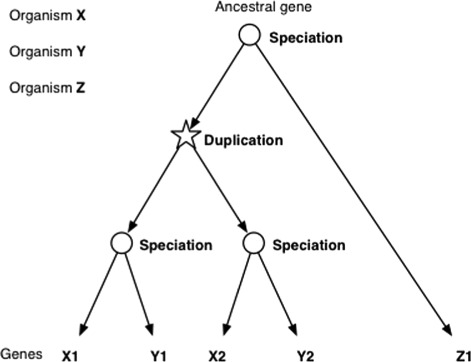
A schematic representation of the evolutionary relations among genes of multiple organisms. The leaf nodes of the tree represent the genes and the internal nodes correspond to evolutionary events. X1 has two ancestral nodes associated with speciation events; one is the last common ancestor with Y1, and the other is common with Z1. Thus, Y1 and Z1 are orthologs of X1. On the other hand, X2 and Y2 are paralogs to X1, since their last common ancestor has a duplication event associated. Likewise, all the pairwise orthology/paralogy relations can be defined according to the strucutre of the given tree

The concepts of orthology and paralogy are defined as specific types of homology [1]; homologs are genes diverged from an ancestral gene, and specifically, orthologs are those diverged by a speciation event, whereas paralogs diverged by a duplication event. Figure 1 shows a schematic representation of evolutionary relations among genes of multiple organisms, which exemplifies orthology/paralogy. Orthologs are usually more conserved in biological functions than paralogs; thus, the orthology relation is particularly useful in transferring the biological knowledge of model organisms to organisms with newly sequenced genomes. Whereas the homology relations are basically calculated in a pairwise perspective, they are often represented as a cluster of homologs. Likewise, an ortholog cluster stands for a group of genes derived from a speciation event, and a paralog cluster for a group of genes derived from a duplication event. Ortholog/paralog clusters can be structured in a form of nested hierarchies, reflecting their evolutionary histories. A simple example of hierarchical clusters can be seen in Fig. 1.

The Quest for Orthologs (QfO) Consortium has identified more than forty resources about orthology (http://questfororthologs.org/orthology_databases), which reflect different scopes of information management in the orthology field. Many of these databases store information about prediction of gene evolutionary relations and there is a diversity of objectives for these databases. There is heterogeneity in how data are stored and provided by these databases. For example, InParanoid [2] stores orthology relations between two species, whereas OrthoMCL [3] and MBGD [4] stores ortholog groups among multiple genomes. OMA [5] provides various types of orthology relations including pairwise orthologs and hierarchical orthologous groups. Traditionally, each resource has used its own representation format based on tabular files, but this community has developed in the last years the OrthoXML format [6] to standardize the representation of orthology data. OrthoXML permits the comparison and integration of orthology data from different resources within the orthology community. However, only a limited number of databases have provided their content using OrthoXML so far.

In recent years, the Semantic Web formats have been used for representing orthology data. OGO [7] was created with the purpose of providing an integrated resource of information about genetic human diseases and orthologous genes. OGO integrated information from orthology databases such as InParanoid or OrthoMCL, plus OMIM [8]. This resource developed an OWL ontology for representing the domain knowledge. More recently, RDF has been used to share the content of the Microbial Genome Database for Comparative Analysis (MBGD) [9]. This resource also developed an OWL ontology for representing the domain knowledge, called OrthO, which had similar concepts to the OGO ones, despite being developed independently.

The report of the 2013 QfO meeting [10] identified a series of aspects about semantics that have been the key drivers of our activities: (1) the orthology community should use shared ontologies to facilitate data sharing; (2) exploiting automated reasoning should be beneficial for the QfO consortium. In this paper, we describe the construction of the Orthology Ontology by reusing the existing related ontologies and we explain how we integrated the existing orthology datasets using the Semantic Web technologies. This work provides a step forward towards the standardization in the orthology community.

## Construction and content

### Construction of the Orthology Ontology

As the first principle, we followed the best practices in ontology engineering: reusing the existing ontologies to facilitate interoperability across biomedical domains; and designing the ontology with a modular perspective, so that different modules of the ontology are created in different sub-taxonomies of the ontology, and the classes from different modules are connected through object properties. The second principle is to define local URIs for basic terms of the domain. In case that equivalent classes are found in the reused ontologies, such equivalency is stated by means of axioms.

Two application-oriented domain ontologies were the starting point for this work, namely, OGO [7] and OrthO [9]. These ontologies provided a basis for discussion and identification of the relevant classes and properties for this domain. Those ontologies already reused some ontologies such as the Relations Ontology (RO) or the NCBI Taxonomy (NCBIT), so these were included in the initial set of candidate ontologies to reuse.

The objective of the Orthology Ontology (ORTH) is to become the reference in the orthology domain and across the biological domains, so it must be beyond the application-oriented ontologies. In order to facilitate interoperability, we decided to search for existing ontologies which could play such interoperability enabler role. We searched repositories such as BioPortal [11], Ontobee [12] and AberOWL [13], and identified ontologies containing classes and properties for the entities identified in our analysis. This list of ontologies is described next:

- Comparative Data Analysis Ontology (CDAO)^1^ [14]: Classes and properties relevant for evolutionary studies.
- Relations Ontology (RO) ^2^ [15]: Collection of biomedical properties to support standardization across biomedical ontologies.
- Homology Ontology (HOM)^3^ [16]: Classes related to homology.
- Sequence Ontology (SO)^4^ [17]: A set of classes and properties to define sequence features used in biological sequence annotation.
- Ontology of Genes and Genomes (OGG)^5^ [18]: Classes and properties to represent relations among genes, genomes and organisms.
- Protein Ontology (PR)^6^ [19]: Protein-related entities, including evolutionary relations between proteins.
- Semanticscience Integrated Ontology (SIO)^7^ [20]: Classes and properties for rich description of biomedical objects and processes.
- NCBI Taxonomy (NCBIT)^8^ [21]: Curated classification and nomenclature for all the organisms.
- Clusters of Orthologous Groups Analysis Ontology (CAO)^9^ [22]: Classes to support the Clusters of Orthologous Groups enrichment method using Fisher’s exact test.

The HOM and the CAO ontologies were discarded for different reasons. On the one hand, we found that the RO properties were more appropriate than the HOM classes for describing relations between biological sequences. On the other hand, the CAO was found too specific and presented overlaps with other ontologies that we consider more relevant for our goal. OGG and PR were not used because their classes of interest are covered by other ontologies. Next, we enumerate the ontologies selected for reuse:

- The RO is the main reference for the properties included in the ORTH.
- The CDAO provides classes for representing evolutionary events such as speciation and duplication, which are fundamental for the orthology domain. Besides, it defines classes and properties for representing the tree, which is a hierarchical structure widely used to represent evolutionary relations. This ontology is reused specially for evolution-oriented entities.
- The SIO provides classes and properties that describe biomedical objects and processes, therefore it is a more general ontology than the CDAO. This is why we have used it as a reference for the general biomedical entities.
- The SO provides classes related to biological sequences, some of which are of interest for the orthology domain: biological region, gene and protein.
- The NCBIT provides the classes for the species associated with the biological sequences.

Besides, it must be taken into account that some SIO properties are equivalent to RO ones. For those cases, we have selected the SIO one. In summary, we selected to reuse SIO and RO for a more general content, CDAO for the evolution-oriented content, SO for the biological sequence types, and NCBIT for the organisms. The above described ontologies provide the biological background knowledge for the orthology domain. Besides, the ORTH reuses other vocabularies:

- dcterms^10^: It includes the metadata terms maintained by the Dublin Core Metadata Initiative. We reused properties such as identifier.
- VoID^11^: RDF Schema vocabulary for expressing metadata about RDF datasets. ORTH needs to represent orthology databases, so the properties and classes representing datasets and membership to them are reused.

### The content of the Orthology Ontology

The Orthology Ontology is available at http://purl.org/net/orth in OWL format. In our model, evolutionary information among sequences are primarily represented as membership of the sequences to clusters of homologs, orthologs or paralogs. Note that the pairwise orthologs/paralogs can be obtained by traversing the tree structure of the clusters. When we see the example shown in Fig. 1, each gene represented by the leaf node belongs to ancestral nodes corresponding to clusters of orthologs or paralogs, from which pairwise orthology/paralogy can be extracted.

**Fig. 2:**
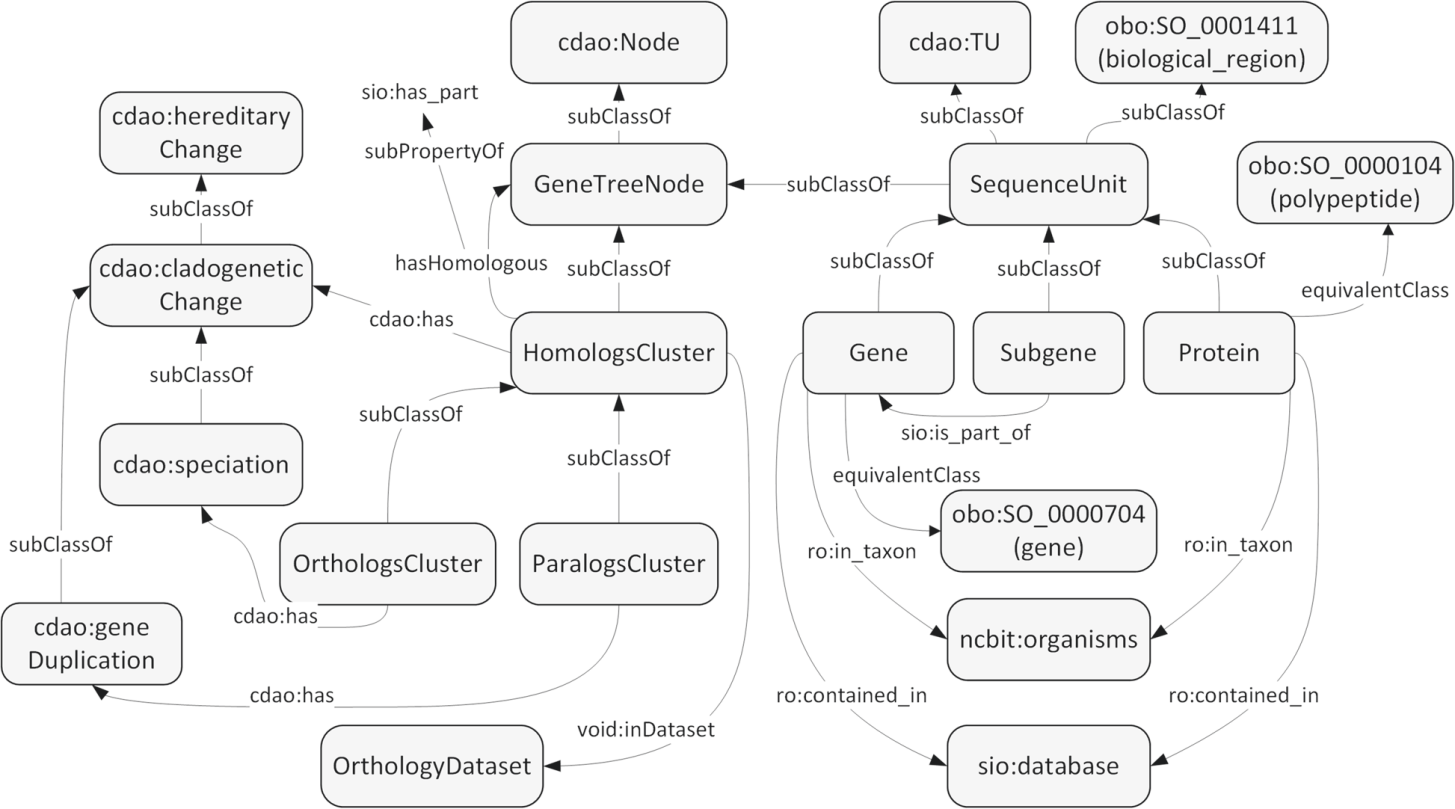
The core classes and properties of the Orthology Ontology. The classes are represented as boxes and the properties as arrows. The prefixes cdao, sio, ro, ncbit and void represent entities reused from the corresponding ontologies. The entities without prefix are defined in the ORTH. On the whole, this figure includes three kinds of classes, each shown in the *left/center/right* parts, respectively: (*left*) classes for evolutionary changes; (*center*) classes for groups of biological sequences holding particular evolutionary relations; and (*right*) classes for biological sequences of interest

Figure 2 shows the core classes and properties included in the ORTH, where three areas can be distinguished as follows. The left side of the figure contains the CDAO module that defines cladogenetic changes, that is, the types of evolutionary events relevant for the orthology domain, such as *cdao:speciation* or *cdao:geneDuplication*.

The central part of the figure describes the main orthology-specific classes from our modeling perspective, that is, the clusters of homologs, orthologs and paralogs, which are represented by means of the classes *HomologsCluster*, *OrthologsCluster* and *ParalogsCluster* respectively. Given that these clusters are usually organized as trees, we have also defined the class *GeneTreeNode*, which is a subclass of *cdao:Node*. Provided that the types of cluster are related to a specific type of cladogenetic change, the property *cdao:has* links the types of clusters with the corresponding cladogenetic changes. Again, we are reusing CDAO content to provide interoperable evolutionary content. Besides, the membership to a given cluster is expressed through the property *hasHomologous*, which is a subproperty of *sio:has_part*, whose inverse property is equivalent to *ro:part _of*. We use this property instead of two hasOrthologous and hasParalogous, because the pairwise relations are obtained by analyzing the tree.

The right side of the figure focuses on the definition of the biological sequences relevant for the orthology domain: genes, subgenes and proteins. These classes are subclasses of *SequenceUnit*, which is a subclass of *cdao:TU*, which represents taxonomic units. The class *Subgene* has been created because of the increasing interest in creating evolutionary analyses of gene subsequences. Hence, its relation with gene has been made explicit through the property *sio:is_part_of*. These classes of the ontology are connected with the central module through the *rdfs:subClassOf* relationship between *SequenceUnit* and *GeneTreeNode*. Genes and proteins, which are defined equivalent to classes in the SO, are linked to *ncbit:organisms* through the property *ro:in_taxon* and to biological databases through *ro:contained_in*.

Although the original terms orthology and paralogy are binary relationships between genes, the ORTH does not include these terms. Instead, the ORTH defines these concepts through the classes *OrthologsCluster* and *ParalogsCluster*. This is because the relationships orthology and paralogy are not transitive [23], and the tree (or hierarchical clustering) representation as shown in Fig. 1 is a better representation for these relationships among multiple genes. In fact, any pairwise orthology/paralogy relation can be extracted from this representation using a query as shown in the next sections. The content has been modeled with the aim of providing an appropriate degree of axiomatization. For instance, we have mentioned that classes such as *OrthologsCluster* and *ParalogsCluster* are associated with the corresponding evolutionary event through an object property. For example, the semantically equivalent definition for an *OrthologsCluster* according to our ontology and thanks to the axiomatization would be a cluster of homologs whose event associated is speciation. This corresponds to the OWL axiom*orth:OrthologsCluster rdfs:subClassOf(orth:HomologsCluster andcdao:has only cdao:CDAO_0000121)*and in SPARQL as follows:?cluster rdf:type/rdfs:subClassOf*orth:HomologsCluster.?cluster cdao:has cdao:CDAO_0000121.

### Ontology metrics

The current version of the ORTH has a core that consists of 21 classes, 14 object properties, 5 datatype properties and 142 axioms, whereas the whole knowledge framework, that is, with the imported ontologies, consists of 4613 classes, 806 object properties, 15 datatype properties and 43140 axioms.

We have applied the OQuaRE framework [24] to evaluate the quality of the ontology produced. With this framework a series of metrics can be calculated, providing scores in the range 1 (lowest) to 5 (highest) for the OQuaRE quality characteristics. Table 1 shows the OQuaRE scores for the ontology with the imported, reused ontologies (complete) and the ontology without the imports (no imports). The ontology also passed successfully the test of the OOPS! Ontology Pitfall Scanner [25].

**Table 1:** Scores of the OQuaRE quality characteristics for the ORTH

ORTH	Structural	Funct. adequacy	Compatibility	Maintainability	Operability	Reliability	Transferability
Complete	4.5	4.56	3.0	3.97	4.33	3.12	4.0
No imports	4.0	4.03	4.25	4.09	3.66	3.0	4.0

The first row shows the scores for the OWL file including the imported ontologies, whereas the second row shows the ones for the ontology without the imported ones

## Applying the ORTH to orthology datasets

In this section we illustrate through an example how the availability of ORTH can benefit the exploitation of orthology data. Experiences have been gained with OMA and InParanoid [26], which have been recently extended to TreeFam [27] in the context of the BioHackathon 2015. Let us suppose that we are doing some research on serum amyloid A1 (SAA1) protein which is known as an inflammatory marker, and that we are interested in finding out if this human gene has orthologs in mouse, because this could permit to carry out some related research with mice.

In this example we assume the existence of three orthology resources: OMA, InParanoid and TreeFam. The use of the original resources to answer this question would require to perform three queries, one per resource and to process and interpret the set of results knowing how orthology relations are represented in each resource. The ORTH ensures that each data represented has a precise meaning, so the user can focus on interpreting the results. Besides, the use of the ORTH for representing the datasets enables to obtain the results with one, non resource-dependent query. The joint exploitation of the orthology datasets requires (1) generating RDF versions of the datasets; and (2) defining and executing the corresponding queries in SPARQL. Both tasks are described in the next subsections.

### Generation of the RDF datasets

We describe next an example of how the source data are transformed into RDF. Let us consider the information available in InParanoid 8 about *Homo sapiens* - *Mus musculus* orthologs^12^. Table 2 shows fragments of the corresponding OrthoXML file. We use OrthoXML as data schema because it is considered a standard in the orthology community. The *species* tags are used to specify the name of the species, the database from which the genes/proteins are retrieved, and the genes used in this file. For each <*gene* > three attributes are shown: (1) *id*, whose scope is the OrthoXML file; it is the ID used for associating a gene with the corresponding clusters; (2) *protId*, which is the identifier of the protein in the database; and (3) *geneId*, which represents a gene symbol in this example. For example, the gene with id 33162 is the human protein whose UniProt accession number (AC) is P0DJI8 and whose gene symbol is SAA1. In this fragment we can see that it contains genes from humans and mice. After the declaration of species and genes, the OrthoXML file includes the cluster with id 16021, which contains the human genes SAA1 and SAA2 and the mouse genes Saa1 and Saa2. This implies: (1) a many-to-many orthology relation between the human and mouse genes, that is, <(SAA1, SAA2), (Saa1, Saa2) >; and (2) the paralogy relation between the genes of the same species, that is, (SAA1, SAA2) and (Saa1, Saa2).

**Table 2:** Fragments of the InParanoid OrthoXML file that stores orthology relations between human and mouse

<species name="Homo sapiens " NCBITaxId="9606" >
<database name="UniProt"
version="UniProt_Complete_Proteomes_2013_06"
protLink="http://www.uniprot.org/uniprot/">
<genes>
<gene id="33162" protId="P0DJI8" geneId="SAA1"/>
<gene id="33163" protId="P0DJI9" geneId="SAA2"/>
</genes>
</species>
<species name="Mus musculus " NCBITaxId="10090">
<database name="UniProt"
version="UniProt_Complete_Proteomes_2013_06"
protLink="http://www.uniprot.org/uniprot/">
<genes>
<gene id="33164" protId="P05366" geneId="Saa1"/>
<gene id="33165" protId="P05367" geneId="Saa2"/>
</genes>
</species>
<orthologGroup id="16021">
<geneRef id="33162"/>
<geneRef id="33163"/>
<geneRef id="33164"/>
<geneRef id="33165"/>
</orthologGroup>

**Fig. 3:**
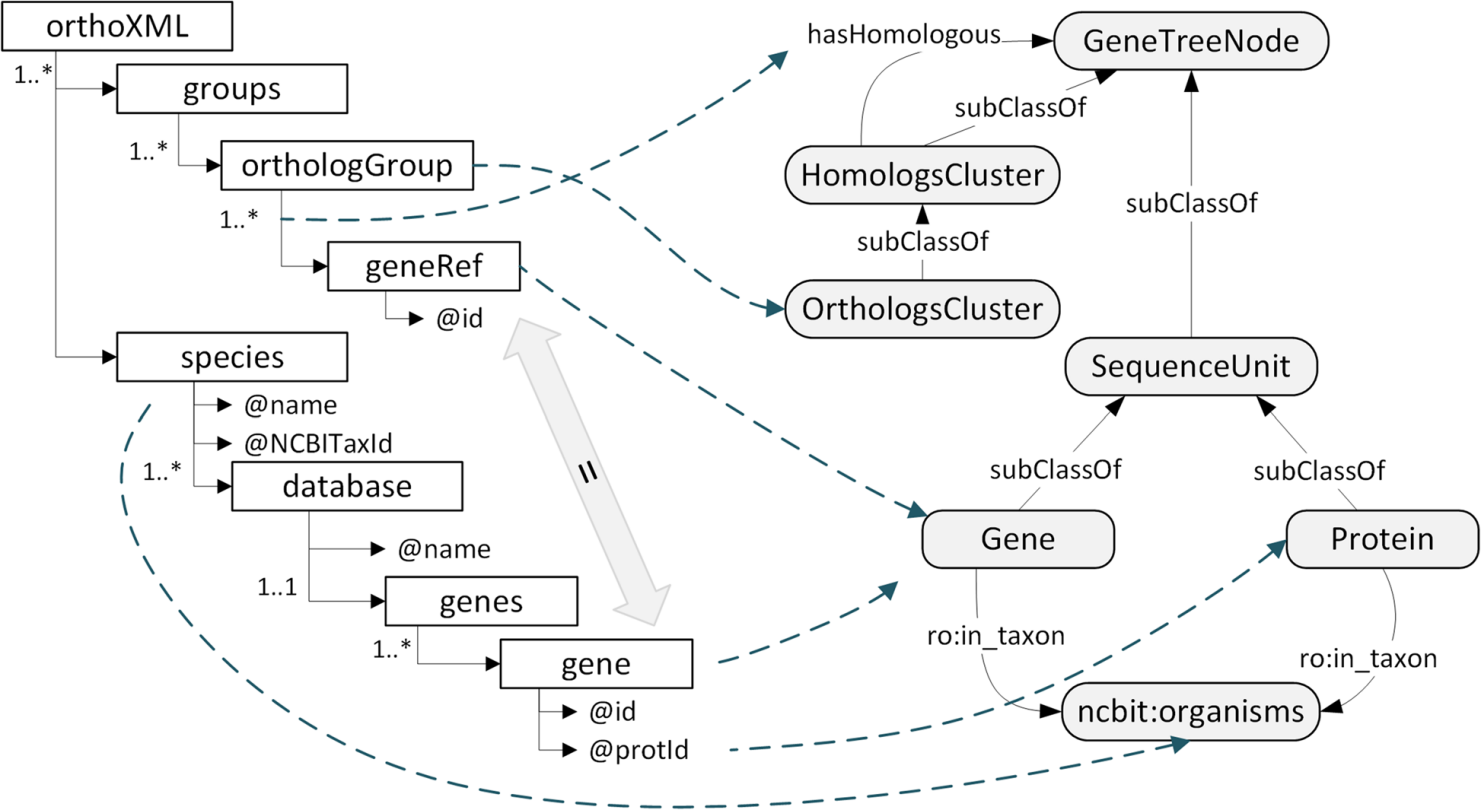
Excerpt of the mapping from the OrthoXML schema (*left*) to the ORTH (*right*). The dashed lines represent the concrete mappings from OrthoXML entities to ORTH classes or properties

The RDF representation of the XML content using the ORTH is obtained by (1) mapping the OrthoXML format to the ORTH; and (2) applying the mappings to the data. Briefly speaking, the mappings associate entities and attributes of the OrthoXML schema with *owl:Class*, *owl:DatatypeProperty* and *owl:ObjectProperty* defined in the ORTH. The mapping file can be found at https://github.com/qfo/OrthologyOntology. An example of the mapping for the clusters of orthologs is shown in Fig. 3, where the left part shows the OrthoXML schema and the right side shows the ORTH schema. There, we can see that the entity *orthologGroup* is mapped to the class *OrthologsCluster* and that the membership of a gene to an *orthologGroup*, which is represented by the link between *orthologGroup* and *geneRef*, is mapped to the object property *hasHomologous*. Consequently, for each *geneRef* included in an *orthologGroup*, the corresponding triple is obtained in the form of *OrthologsCluster hasHomologous Gene*.

Technically speaking, the generation of the RDF datasets is supported by the SWIT tool ^13^. SWIT is able to generate RDF and OWL content by applying the mapping rules to the OrthoXML versions of OMA, InParanoid and TreeFam. Besides, SWIT uses automated reasoning to ensure that only logically consistent content is transformed. This means that the data instances inconsistent with the axioms of the ORTH are not transformed into RDF or OWL. Table 3 shows the RDF triples generated for describing the orthologous group 16021.

**Table 3:** RDF triples for the cluster of orthologs 16021

orth_data:orthologsCluster_16021 rdf:type orth:OrthologsCluster.
orth_data:orthologsCluster_16021 void:inDataset orth_data:
orthologyDataset_InParanoid.
orth_data:orthologsCluster_16021 dcterms:identifier "16021".
orth_data:orthologsCluster_16021 orth:hasHomologous orth_data:
gene_33162.
orth_data:orthologsCluster_16021 orth:hasHomologous orth_data:
gene_33163.
orth_data:orthologsCluster_16021 orth:hasHomologous orth_data:
gene_33164.
orth_data:orthologsCluster_16021 orth:hasHomologous orth_data:
gene_33165.

The RDF datasets generated from InParanoid 8, OMA hierarchical orthologous groups (Sep 2014) and TreeFam 9 are available on our website, and contain 8798758 genes from OMA, 1713180 genes for *Homo sapiens* orthologs and 1367940 genes for *Mus musculus* orthologs from InParanoid, and 1376021 genes from TreeFam. Overall, the complete dataset has over 2 billion triples.

### Exploitation of the RDF datasets

Here we assume the existence of an RDF repository with the data from the three resources, which has been generated as described in the previous section. In case of using three distinct RDF repositories, the SPARQL queries should be adapted by including the corresponding SERVICE clauses. Given that we are interested in retrieving the orthologs of the human gene SAA1 in mouse, answering this query requires to extract pairwise orthology relations betwen the human gene and mouse genes from the repository. The ORTH associates each gene with (hierarchical) clusters of homologs to which it belongs and each cluster with an evolutionary event. Thus, extracting the pairwise orthologs means searching for such genes that are members of the same cluster as the human gene SAA1 belongs to and the last ancestor cluster is a cluster of orthologs (i.e., it has a speciation event associated).

Such description can be expressed as the SPARQL query shown in Table 4. This query can extract orthologous pairs by identifying their last common ancestors; it extracts pairs of genes (*?gene1* and *?gene2*), that are the descendants of respective two distinct nodes of the tree (*?tree_node1* and *?tree_node2*) whose common parent (*?common_ancestor*) is a cluster of orthologs. The tree-based cluster analysis is facilitated by the use of the single property *hasHomologous*, which is transitively used when the symbol * is attached to it. The FILTER and VALUES clauses serve to define the gene and organisms of interest for the query, meaning that it could be used as a template for finding pairwise orthologs for any given gene; only the VALUES clause would have to be modified.

**Table 4:** A sample query for getting the orthologs of a given gene

SELECT ?gene ?species ?database WHERE {
?common_ancestor a orth:OrthologsCluster.
?common_ancestor ort:hasHomologous ?tree_node1.
?common_ancestor orth:hasHomologous ?tree_node2.
?common_ancestor void:inDataset ?dataset.
?dataset orth:hasSource ?database.
?tree_node1 orth:hasHomologous* ?gene1.
?tree_node2 orth:hasHomologous* ?gene2.
?gene1 a orth:Gene.
?gene2 a orth:Gene.
?gene1 obo:RO_0002162 ?species1.
?gene2 obo:RO_0002162 ?species2.
?gene1 dcterms:identifier ?id.
?gene2 dcterms:identifier ?gene.
?species2 rdfs:label ?species.
bind(str(?id) as ?str_id)
FILTER (?tree_node1 != ?tree_node2 && ?species1 != ?species2)
VALUES (?str_id ?species1 ?species2) {(“SAA1” ncbit:9606 ncbit:10090)}
}

In this example, the mouse (ncbit:10090) orthologs of the human (ncbit:9606) gene SAA1 are retrieved. obo:RO_0002162 stands for the property in_taxon

The results of the query are shown in Table 5. We can see that there is a one-to-many relation between the human gene SAA1 and its orthologs in mouse (SAA1, SAA2, SAA3) and that two resources contain the results. The relationship with SAA1 and SAA2 is supported by the two resources, and only OMA proposes the one for SAA3. It should be noted that the predicted orthology relation is generally reliable in the case of one-to-one, but conflicts between methods often happen when the relationship is not one-to-one. More details, including sample queries that exploit the three resources, can be found at https://github.com/qfo/OrthologyOntology.

**Table 5:** Results of the query shown in Table 4 for a repository that integrates InParanoid and OMA

Gene	Species	Database
SAA1	*Mus musculus*	OMA
SAA3	*Mus musculus*	OMA
SAA2	*Mus musculus*	OMA
Saa1	*Mus musculus*	InParanoid
Saa2	*Mus musculus*	InParanoid

## Utility and discussion

### Potential applications

The development of the Orthology Ontology enables a series of activities that will show progress in how orthology data are represented and exploited.

1) Linked Orthology Data to promote interoperability. Many biological databases include information about orthology relations, which derive from different orthology resources created by different methods. The ORTH vocabulary can be used to generate shareable RDF datasets that could be queried by biomedical informatics tools. An initial research on how the ORTH can drive the transformation of orthology databases in OrthoXML is reported in this work. The development of a Linked Data API for ORTH datasets would permit to standardize a series of methods that would return data from different resources preserving the meaning of the entities, so promoting the standardization of the orthology data obtained from different resources such as UniProt or Ensembl.

2) Meta-predictor of orthology for better prediction of biological functions. Predicting biological functions is likely to be the most widespread application of orthology resources. The availability of the ORTH and the existence of RDF orthology datasets based on the ORTH will facilitate the development of methods for improving orthology prediction by exploiting the predictions of many of the existing orthology resources, which can improve the function prediction by orthology relations. The potential of this meta-approach will be reinforced by the standardization effort of the orthology content.

3) Migration of existing resources. Data migration of existing orthology resources described by previous ontologies such as OrthO is also necessary and can be done with the support of the results of the present work. We provide information that helps the ontology users catch up the evolution of the ontologies and work on the data migration. As an example, we have summarized the term-by-term correspondence between previous ontologies (OGO, OrthO) and the current ORTH (see https://github.com/qfo/OrthologyOntology). This table will help replace the previous ontologies with the current ontology. In fact, we have already replaced the OrthO ontology of MBGD database (http://mbgd.genome.ad.jp/sparql) according to this table. The replacement was straightforward as the current ontology covers the previously used concepts. Toward the ontology standardization, not the distinct researcher’s but a community-oriented approach is crucial. The current ontology with enhanced consensus and semantics will be more suitable than previous ones for standardization and further application of orthology resources.

### Data integration issues

ORTH-based data integration can be carried out following two main approaches: links or warehouse. In the link-based approach, there would be one RDF dataset using the ORTH vocabulary per orthology resource, and it is the data integration strategy used in projects such as Bio2RDF [28]. The application of the link-based strategy to OMA and InParanoid for the SAA1 example would produce one instance of SAA1 in each repository. Both instances could have the same URI (e.g., http://identifiers.org/hgnc.symbol/SAA1) or different ones, which would depend on the decision made by the data providers, since gene nomenclature is well maintained only for limited species. In this latter case, *owl:sameAs* links should be defined to identify that they refer to the same gene.

The warehouse approach stores the whole dataset in the same repository, and it is the approach followed in projects such as OGOLOD [29]. This approach requires to be able to identify which instances from the different datasets refer to the same gene or protein, which is easy to find in case shared identifiers are used, but difficult otherwise. The application of the warehouse strategy to OMA and InParanoid for the SAA1 example would produce one instance of SAA1, which would integrate the content from OMA and InParanoid.

In the current work, we have followed a warehouse approach using resource-oriented URIs, with the objective of studying and make visible the data integration issues that would impede the orthology community to have semantically interoperable datasets even with the availability of the ORTH. We wanted to test to what extent the availability of the ORTH and the definition of a common transformation process could help, and what additional work should be done. The results obtained are three datasets that use the same knowledge framework, which can be jointly queried, which is one of the contributions of the present work, since those datasets could not be jointly queried to date. This means that there is one instance of SAA1 for the gene from each resource. Besides, the proper integration of data has not been carried out through links.

The heterogeneity at the identifier level is an important issue in the application of the ORTH. Table 6 shows that different identifiers are used by the three resources: InParanoid, OMA and TreeFam. OMA uses local identifiers for the proteins; InParanoid uses the UniProt AC for proteins and the gene symbol for genes; and the identifiers used in TreeFam depend on the database used for the corresponding species (e.g., Ensembl, FlyBase, WormBase). We think that the generation of highly interoperable RDF orthology datasets following the link-based approach would require the data providers to support with a mapping service for generating the corresponding links or to use common reference identifiers. One practical approach is that each database provider should provide cross-references to at least one of the common public sequence databases, such as RefSeq and UniProt, in an unambiguous form.

**Table 6:** Summary of sequence sources and identifiers used in three different orthology resources

Orthology resource	Database source	Protein ID	Gene ID
OMA	Multiple sources	OMA ID	Gene symbol^a^
InParanoid	UniProt	UniProt AC	Gene symbol^a^
TreeFam	Multiple sources	Multiple sources	Multiple sources

^a^The gene symbol is used for model organisms, including human and mouse, but this is not the case for other organisms in general

Note that the conventions for writing gene names are not always normalized. As seen in the search results in Table 5, OMA dataset returned SAA1, which derives from the UniProt mnemonic we used, while InParanoid returned Saa1 as the name of the gene. The latter would be the correct one according to the nomenclature for *Mus musculus*. This can be technically solved in an easy way, because SPARQL queries can ignore the case, but we believe that standardized names, or more preferably, common URIs should be used in integrative analyses.

We are currently representing the data with attribution to the source, as shown in Table 3. Given that the relations proposed by each resource depend on the particular prediction method used, different resources might predict different orthologs. This complementary information will be the input to the meta-predictor of orthology. To resolve conflicting information, one of the simplest ways is to use a majority vote, but the meta-predictor might also be able to use a confidence level if each predictor returns such a value (e.g. bootstrap value) with its prediction. In fact, the OrthoXML specification includes such information, named score, although currently this slot can contain any type of value and further normalization is needed to compare the scores obtained from different resources. Alternatively, the meta-predictor might use authority levels defined for each resource in case conflicting information is found. Conflicts are easier to detect with our approach, since we could find them through queries that exploit the RDF datasets. Since they are predictions, we think that at least reporting conflicts among different methods should be helpful for the researchers to utilize these predictions.

The technical tasks have been done using the SWIT tool, which permits to define which URIs to use for the dataset, and in the present work we decided to use resource-oriented URI instead of a common URI approach due to the aforementioned heterogeneity of identifiers. All these resources provide clusters of orthologs/paralogs, for which local, sequential numbering is used, which means that resource-oriented URIs are needed. SWIT permits to combine resource-oriented and external URIs for the different entities. For instance, we have used the prefix http://identifiers.org/taxonomy/ for the URI of the species for the three resources. SWIT also includes methods for data integration based on the definition of identity conditions, but they are applicable for the construction of one single dataset. Such conditions would permit to merge the data about the same gene name from different resources.

## Limitations and further work

The CDAO has been the ontology reused for many evolution-oriented entities, including the nodes of the hierarchical structure. It might be worth studying whether the complete representation for trees provided by CDAO, including the description by means of nodes and edges, can be useful and practical for our objectives. The engineering of the ontology information also needs to be further evaluated. The ontology quality scores for the current version of ORTH are positive, but the analysis of the quality metrics and characteristics reveals that it can be improved. Such scores will be further analyzed to estimate which parts of the ontology can be improved. The experience with the use and application of the ORTH is still limited, and the structure of the content has been mainly exploited to derive pairwise orthology and paralogy relations from resources that provide data in OrthoXML format. The transformation of OrthoXML datasets has permitted us to detect that the data providers are not using this format in an homogeneous way either. For example, an important piece of information such as the taxonomic range, at which a given cluster is obtained, is expressed in different ways by different resources using OrthoXML user-defined properties. On the one hand, OMA uses a property called TaxRange, whose value is a string (e.g., “Insectivora”). On the other hand, TreeFam uses two properties called taxon_name and taxon_id. TaxRange is equivalent to taxon_name and the value for taxon_id is the NCBI Taxonomy ID. This has forced us to define two different mapping rules to generate the RDF content corresponding to the taxonomic range. The other mapping rules are shared by the three resources. In addition to this, we have found situations in which the gene symbol was not provided for the genes of all the species in a particular OrthoXML dataset. Further exploitation to obtain other relations of interest such as in-paralogy or out-paralogy, and many-to-many relations need to be examined. There is also room for incorporating additional concepts, such as horizontal gene transfer and the corresponding term xenolog [30], which are required to describe prokaryotic gene evolution. Provided that this effort rises from the needs identified in the Quest for Orthologs community, a community-driven evaluation will be performed.

## Conclusions

Orthology relations play a fundamental role in computational comparative analysis, which can provide valuable opportunities to biomedical research. Current orthology databases are heterogeneous in the structure and meaning of the domain entities they use, hampering the data interoperability within the orthology domain and across biomedical domains. The Ontology Orthology can provide a standardized vocabulary for the representation of orthology datasets. Initial efforts have demonstrated the development of shareable datasets in RDF, but further applications need to be developed to maximize the usefulness of the Orthology Ontology.

## Endnotes

^1^http://purl.bioontology.org/ontology/CDAO.

^2^https://github.com/oborel/obo-relations.

^3^http://purl.bioontology.org/ontology/HOM.

^4^http://purl.bioontology.org/ontology/SO.

^5^https://bioportal.bioontology.org/ontologies/OGG.

^6^http://purl.bioontology.org/ontology/PR.

^7^https://code.google.com/p/semanticscience/wiki/SIO.

^8^http://purl.bioontology.org/ontology/NCBITAXON.

^9^http://purl.bioontology.org/ontology/CAO.

^10^http://purl.org/dc/terms/.

^11^http://vocab.deri.ie/void.

^12^http://inparanoid.sbc.su.se/download/8.0_current.

^13^http://sele.inf.um.es/swit.

## Abbreviations

CAO: clusters of orthologous groups analysis ontology; CDAO: comparative data analysis ontology; HOM: homology ontology; NCBIT: NCBI taxonomy; SO: sequence ontology; PR: protein ontology; OGG: ontology of genes and genomes; ORTH: orthology ontology; OWL: web ontology language; RDF: resource description framework; RO: relations ontology; SIO: semanticscience integrated ontology
